# Contemporary and historical oceanographic processes explain genetic connectivity in a Southwestern Atlantic coral

**DOI:** 10.1038/s41598-018-21010-y

**Published:** 2018-02-08

**Authors:** L. Peluso, V. Tascheri, F. L. D. Nunes, C. B. Castro, D. O. Pires, C. Zilberberg

**Affiliations:** 1 Departamento de Zoologia, Instituto de Biologia, Universidade Federal do Rio de Janeiro, Cidade Universitária, Rio de Janeiro, RJ Brazil; 2 Ifremer Centre Bretagne, DYNECO, Laboratoire d’Ecologie Benthique Côtière (LEBCO), 29280 Plouzané, France; 3 Departamento de Invertebrados, Museu Nacional, Universidade Federal do Rio de Janeiro, Quinta da Boa Vista, Rio de Janeiro, RJ Brazil; 4 Instituto Coral Vivo, Rua dos Coqueiros, 87, Parque Yayá, Santa Cruz Cabrália, Bahia Brazil

## Abstract

Understanding connectivity patterns has implications for evolutionary and ecological processes, as well as for proper conservation strategies. This study examined population genetic structure and migration patterns of the coral *Mussismilia hispida*, one of the main reef builders in the Southwestern Atlantic Ocean. For this, 15 sites were sampled along its entire distributional range employing 10 microsatellite *loci*. *M*. *hispida* was divided into five genetically differentiated populations by Structure analysis. Population structure and migration estimates are consistent with present-day oceanographic current patterns, zones of upwelling and historical sea-level changes. The Central Region and Oceanic Islands populations had the highest genetic diversity, were possibly the main sources of migrants for other populations and presented mutual migrant exchange. This mutual exchange and the high diversity of Oceanic Islands, a peripherical population, is highly interesting and unexpected, but can be explained if these sites acted as refugia in past low sea-level stance. This is the first connectivity study in the region using hyper-variable markers and a fine sampling scale along 3,500 km. These results enlighten the population dynamics of an important reef building species and shows how oceanographic processes may act as barriers to dispersal for marine species, providing valuable information for management strategies.

## Introduction

Genetic connectivity studies can provide fundamental information to better understand the ecology and evolution of a species. Estimated patterns usually indicate the population dynamics at contemporary and historical time scales, including the paths, directions and frequency of migration, colonization history and source and sink dynamics^1^. It also leads to better conservation strategies, since natural gene flow patterns can be maintained, ensuring long term conservation given that evolutionary processes are preserved^2^.

Understanding connectivity patterns in marine habitats is not as evident as in land. This is because marine species can have high dispersal potential, due to planktonic life stages, the non-obvious nature of barriers to dispersal in the sea and complex biological and physical factors that shape survival and settlement^3,4^. To evaluate gene flow in marine habitats, genetic markers based on frequency data, such as microsatellites, have shown to be useful for estimating connectivity at ecological timescales^4^ and have increasingly been employed for scleractinian corals’ studies in recent years^5–7^. However, some regions of the world’s oceans receive less attention and their connectivity patterns are still poorly known. For instance, in the Atlantic Ocean the majority of connectivity studies for scleractinian corals have focused on Caribbean populations^5,8,9^, while less is known for corals on the Southwestern Atlantic (SWA), a biogeographic region also known as the Brazilian Province^10^.

In the SWA, coral reef communities are distributed along more than 3,500 km of coastline, including the largest biogenic coral reef formation in the South Atlantic Ocean, the Abrolhos Reef Complex in Bahia State, Brazil^11^. These southern coral communities have low diversity of zooxanthellate scleractinian corals (at least 16 species), but its level of endemicity is higher than 20%^11,12^. Despite its uniqueness, only a few studies have addressed gene flow and genetic diversity of scleractinian corals in this region. While connectivity has been assessed for five species thus far (*Siderastrea stellata*, *S*. *radians*, *S*. *siderea*, *Montastraea cavernosa* and *Favia gravida*^13–15^), these studies have either employed relatively conserved markers or have sampled at low spatial resolution such that current knowledge about coral connectivity in the SWA is limited to restricted locations and to more ancient timescales.

One of the main reef-building corals in the SWA is *Mussismilia hispida* (Verrill 1902), an endemic species that has a broad distributional range, occurring from Maranhão (0°S) to São Paulo States (24°S)^11^. This coral is a broadcast spawner with an annual reproductive cycle and lecithotrophic larvae^16^ and, although no studies have evaluated its pelagical larval duration (PLD), preliminary studies indicate that it lasts approximately 10 days^17^. The time of gamete release for *M*. *hispida* has been estimated in four locations in Brazil and an asynchrony in spawning time was observed among them. Gamete release was estimated to take place between April and June in the Abrolhos Coral Reef Complex (17°S)^16^, while less than 200 km to the north, in Porto Seguro, Bahia (16°S), spawning was reported to occur between August and November^17^. Further south, the time of spawning was estimated to be between February and March at Armação dos Búzios, Rio de Janeiro (22°S)^18^ and it was observed *in situ* to occur in April at Laje de Santos, São Paulo (24°S)^19^. Although population connectivity does not rely solely on gamete dispersal, this asynchrony raises questions about reproductive isolation and gene flow patterns in *M*. *hispida*. Such facts make this species a great model to understand connectivity patterns in the SWA, besides providing means to test the effects of reproduction asynchrony in a wide geographical range (i.e., 3,500 km). Therefore, in this study we aimed to address various questions concerning population connectivity of *M*. *hispida* populations along its distributional range using microsatellite data. We tested the hypotheses that (1) the reproductive asynchrony in this species influences its patterns of population structure; (2) *M*. *hispida* is structured across its distributional range; (3) the main barriers to gene flow coincide with previously reported biogeographical breaks in the SWA; and (4) that the gene flow directions agree with the major ocean currents in the region.

## Results

### *Loci* characterization

**Figure 1:**
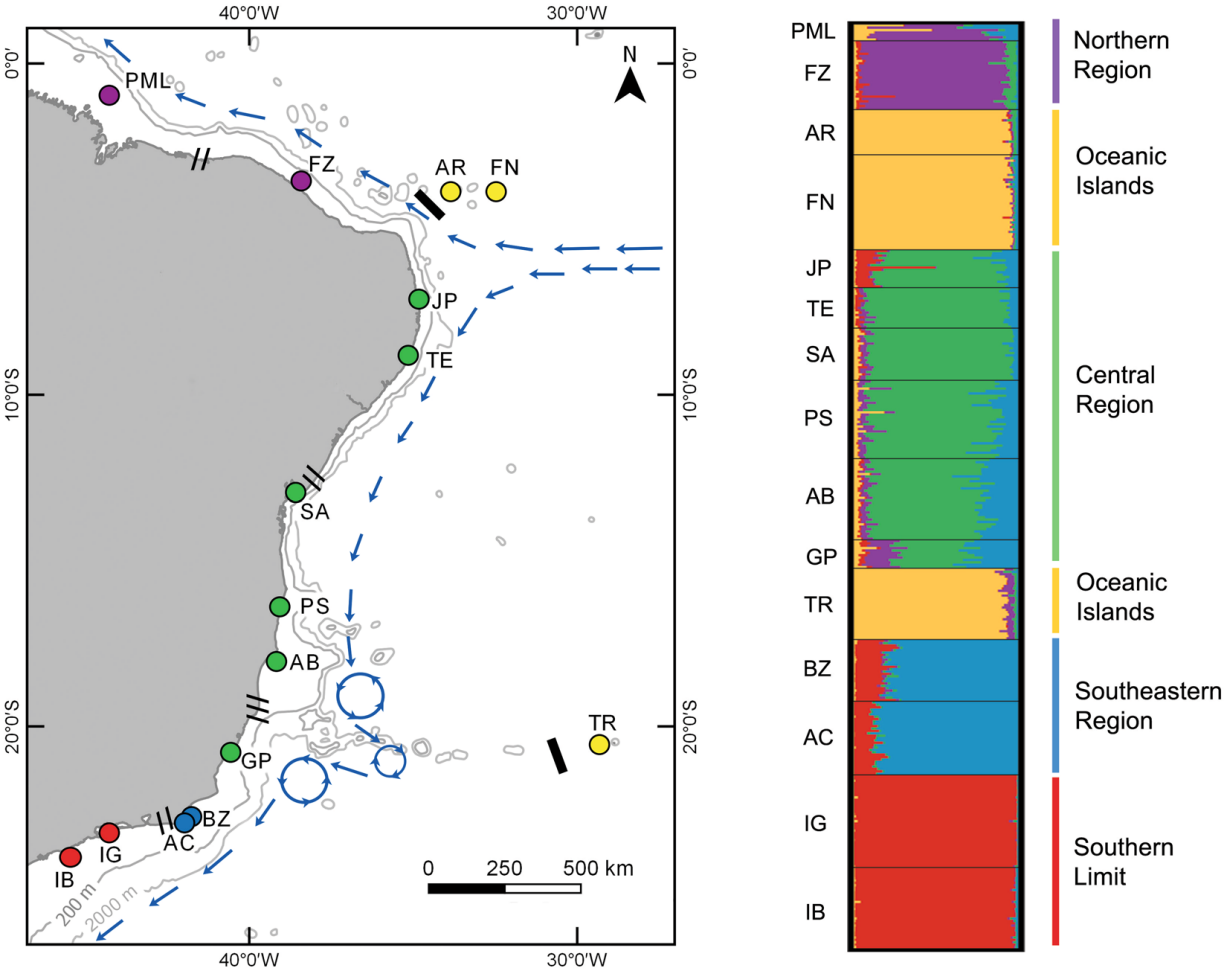
Sampling sites of *Mussismilia hispida* across its distributional range and its population subdivisions. The sampling locations on the map and on the bar plot are abbreviated as in Table 1. On the map, blue arrows indicate an approximation to the major oceanic currents directions^29,57,58^. Possible barriers to gene flow according to Spalding *et al*.^31^ (//), to Floeter *et al*.^32^ (**///**) or both (black rectangles) are indicated. The bar plot shows the average probability of membership of each sample (vertical bars) in each population (colours) for *K* = 5, from 10 iterations and with sampling location as prior. Colours of sampling locations on the map corresponds to the populations’ colours from the bar plot. The map was generated using QGIS 2.8.1 (http://qgis.osgeo.org) and edited using Adobe Photoshop CS6 13.0.1.

A total of 391 samples of *Mussismilia hispida* were genotyped for 13 microsatellite *loci* (Table 1) from fifteen sites across the Southwest Atlantic Ocean (Fig. 1). From these, only two pairs of samples from IB displayed identical *multilocus* genotypes, thus, one individual of each pair was removed. The Mhi24 and Mhi27 *loci* were excluded because they presented unreliable genotypes across the majority of samples. No *loci* displayed linkage disequilibrium and only Mhi4 had evidence of null alleles for fourteen out of fifteen locations. Therefore, this *locus* was also removed, leaving 10 *loci* for the remaining analyses. The number of alleles per *locus* varied from 9 to 58 with an average of 28.5 alleles (Table 2). The *loci* Mhi16 and Mhi17 had the lowest values of allelic richness (Table 2). The majority of *loci* had high values of observed heterozygosity (>0.4), except Mhi16 and Mhi17 (<0.2) (Table 2).

**Table 1:** Sampling site information and genetic diversity per site for *Musssimilia hispida*. Site names abbreviations are used in figures and text.

Sampling site	Code	N	Latitude	Longitude
Parcel do Manuel Luís	PML	7	−0.8727	−44.2609
Fortaleza	FZ	29	−3.5976	−38.4076
Rocas Atoll	AR	19	−3.8668	−33.8021
Fernando de Noronha	FN	40	−3.8542	−32.4453
João Pessoa	JP	16	−7.1129	−34.8130
Tamandaré	TE	17	−8.7579	−35.0859
Salvador	SA	22	−12.9440	−38.5139
Porto Seguro	PS	33	−16.4161	−38.9814
Abrolhos	AB	34	−17.9669	−39.1978
Guarapari	GP	12	−20.7114	−40.5083
Trindade Island	TR	30	−20.5017	−29.3460
Armação dos Búzios	BZ	26	−22.7391	−41.8743
Arraial do Cabo	AC	31	−22.9673	−42.0151
Ilha Grande	IG	39	−23.1469	−44.3217
Ilhabela	IB	34	−23.8706	−45.4406

Number of samples per site (N) and sites approximate GPS coordinates are given (WGS84 standard coordinate system).

**Table 2:** Per *locus* statistics for the 10 microsatellite markers used for *Mussismilia hispida*.

*Locus*	Na	A_1_	A_2_	Ho	Hs	Ht	G_IS_	G_ST_
Mhi1	28	8.175	13.767	0.510	0.669	0.782	0.238	0.144
Mhi2	50	10.676	17.480	0.721	0.841	0.908	0.142	0.073
Mhi14	15	6.578	8.551	0.645	0.762	0.809	0.153	0.058
Mhi16	14	3.317	5.646	0.183	0.229	0.235	0.203	0.022
Mhi17	9	3.259	4.277	0.291	0.323	0.352	0.096	0.083
Mhi18	58	9.743	17.696	0.697	0.790	0.823	0.118	0.041
Mhi20	30	9.291	16.128	0.666	0.758	0.852	0.121	0.111
Mhi21	37	8.337	13.289	0.475	0.737	0.840	0.355	0.122
Mhi23	21	7.819	12.224	0.622	0.735	0.833	0.154	0.119
Mhi26	23	8.750	12.460	0.752	0.834	0.885	0.098	0.057
Mean	28.5	—	—	0.556	0.668	0.732	0.167	0.088

Mean values for total number of alleles (Na), mean allelic richness based on 14 sampling sites (A_1;_ site PML was excluded due to low sample size), mean allelic richness based on the five populations as defined by Structure analysis (A_2_), observed heterozygosity (Ho), heterozygosity within populations (Hs), total heterozygosity (Ht) and Nei’s interbreeding coefficient (G_IS_) and fixation index (G_ST_).

### Genetic diversity

The expected heterozygosity of each *locus*, number of alleles per locality and allelic richness show high values of genetic diversity, with southern localities having the lowest values for the majority of *loci* (Supplementary Tables S1 and S3). Accounting all *loci*, expected heterozygosity varied from 0.49 to 0.80. Most locations had heterozygosity deficiencies compared to what was expected under the Hardy-Weinberg Equilibrium, as demonstrated by the significant F_IS_ indexes, except from FZ, AR and IB (Supplementary Table S3). These estimates show similar patterns when populations are defined by genetic structure analyses (Supplementary Table S4). A decrease in diversity is seen in southern populations and all populations show deviation from the Hardy-Weinberg Equilibrium when accounting all *loci* (Supplementary Tables S2 and S4).

### Population genetic structure

**Figure 2:**
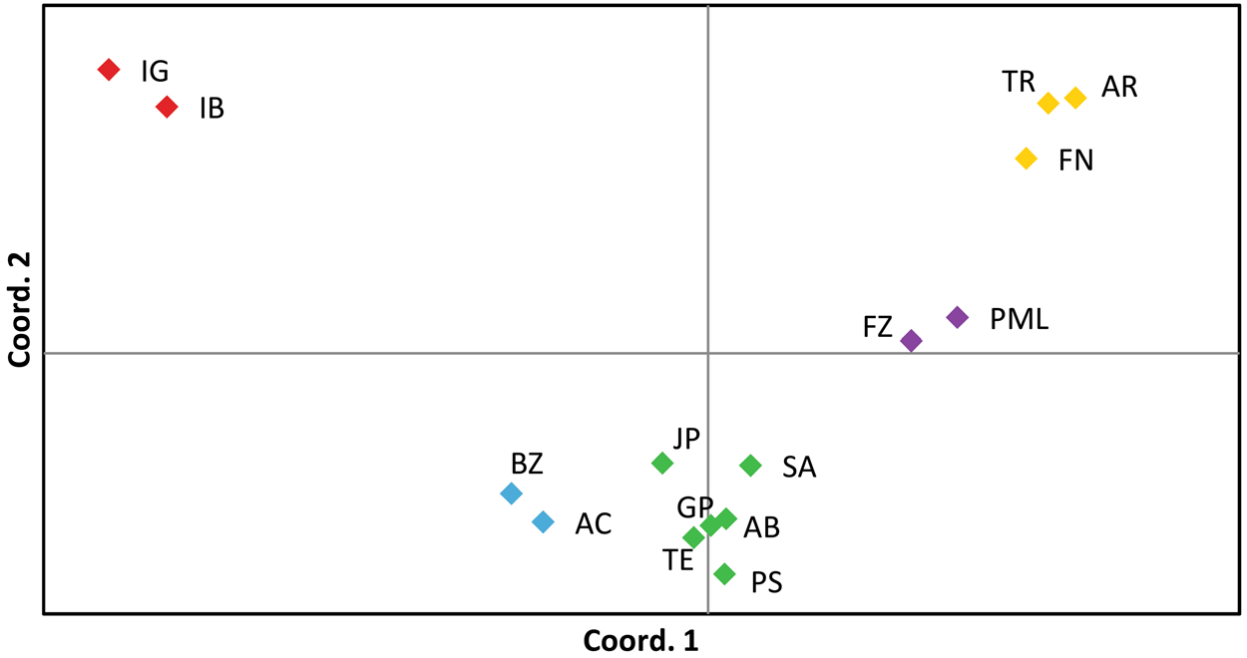
Principal coordinates analysis of the F_ST_ values between localities. Colours correspond to populations of *Mussismilia hispida* as defined by Structure analysis: Northern Region (purple), Oceanic Islands (yellow), Central Region (green), Southeastern Region (blue) and Southern Limit (red). Sampling localities are abbreviated as in Table 1.

The majority of pairwise F_ST_ values were significant, indicating the presence of genetic structure between locations. Overall, the highest F_ST_ values found were between the two southernmost sites (IG and IB) and all the other sites, while between these two localities, although significant, the F_ST_ value was relatively low (Table 3). Principal coordinates analysis (PCoA) of F_ST_ values shows four to five clusters, with axis 1 and 2 explaining 57,6% and 14,9% of the variation, respectively (Fig. 2). These results show that gene flow is high between some localities, usually the ones geographically nearest, and that IG and IB are the most genetically differentiated localities (Fig. 2).

**Table 3:** Pairwise fixation index (F_ST_) between sampling sites of *Mussismilia hispida* along the Southwest Atlantic.

	FZ	AR	FN	JP	TE	SA	PS	AB	GP	TR	BZ	AC	IG	IB
PML	0.018	**0**.**044**	**0**.**035**	**0**.**045**	**0**.**057**	**0**.**054**	**0**.**047**	**0**.**032**	**0**.**050**	**0**.**048**	**0**.**098**	**0**.**089**	**0**.**264**	**0**.**231**
FZ		**0**.**056**	**0**.**044**	**0**.**048**	**0**.**041**	**0**.**071**	**0**.**042**	**0**.**045**	**0**.**044**	**0**.**049**	**0**.**094**	**0**.**094**	**0**.**224**	**0**.**189**
AR			0.007	**0**.**080**	**0**.**087**	**0**.**086**	**0**.**079**	**0**.**072**	**0**.**069**	**0**.**031**	**0**.**141**	**0**.**129**	**0**.**279**	**0**.**242**
FN				**0**.**066**	**0**.**074**	**0**.**083**	**0**.**066**	**0**.**054**	**0**.**061**	**0**.**026**	**0**.**112**	**0**.**107**	**0**.**246**	**0**.**214**
JP					−0.001	**0**.**041**	**0**.**020**	**0**.**008**	0.006	**0**.**072**	**0**.**045**	**0**.**034**	**0**.**148**	**0**.**118**
TE						**0**.**033**	0.003	**0**.**013**	−0.005	**0**.**079**	**0**.**047**	**0**.**049**	**0**.**166**	**0**.**127**
SA							**0**.**037**	**0**.**040**	**0**.**036**	**0**.**090**	**0**.**087**	**0**.**093**	**0**.**184**	**0**.**159**
PS								0.005	0.007	**0**.**086**	**0**.**046**	**0**.**055**	**0**.**174**	**0**.**138**
AB									0.008	**0**.**072**	**0**.**032**	**0**.**035**	**0**.**162**	**0**.**131**
GP										**0**.**068**	**0**.**041**	**0**.**037**	**0**.**183**	**0**.**139**
TR											**0**.**129**	**0**.**123**	**0**.**265**	**0**.**225**
BZ												0.002	**0**.**124**	**0**.**102**
AC													**0**.**137**	**0**.**118**
IG														**0**.**014**

Bold numbers indicate significant values after Bonferroni correction (p < 0.003). Sampling sites are abbreviated as in Table 1.

The isolation by distance analysis showed significant but weak correlation between genetic distance and geographic distance (R^2^ = 0.19, p = 0.003). When the Oceanic Islands were excluded from the analysis, since they had high geographic distances compared to all other localities, the correlation remained weak and significant (R^2^ = 0.17, p = 0.030).

The Structure analysis indicated that the most probable number of populations (*K*) was five. For LnPD and Delta K values, see Supplementary Fig. S1. The five populations are: Northern Region (PML and FZ), Oceanic Islands (AR, FN and TR), Central Region (JP, TE, SA, PS, AB, and GP), Southeastern Region (AC and BZ) and Southern Limit (IG and IB) (Fig. 1). Smaller values of *K* showed which sites were the most differentiated (Supplementary Fig. S2). What is evident from these results, particularly with the *K* = 3–5 plots, is that the Oceanic Islands and Southern Limit have less admixture than the remaining populations (Supplementary Fig. S2). Structure analyses with higher values of *K* only showed further subdivision when *K* = 7 (Supplementary Fig. S2), where TR splits from the other two Oceanic Islands. Although *K* = 7 also appears to be supported by this analysis given its LnPD value (Supplementary Fig. S1), we believe *K* = 5 is more appropriate since it is also corroborated by the Fst PCoA (Fig. 2), not all seven populations can be defined when *K* = 7 (Supplementary Fig. S2) and no substructure can be seen when we ran a Structure analysis considering only the Oceanic Islands sites. This analysis showed that the most probable *K* was two, but the barplot for this *K* did not reveal a clear pattern, as all individuals had approximately 50% of probability of belonging to either population (Supplementary Fig. S3). Therefore, we believe *K* = 5 is the best inference given this dataset. To further confirm this, the existence of substructure was tested for the other four populations, but results showed that the most probable *K* was one for all of them.

### Migration pattern

**Figure 3:**
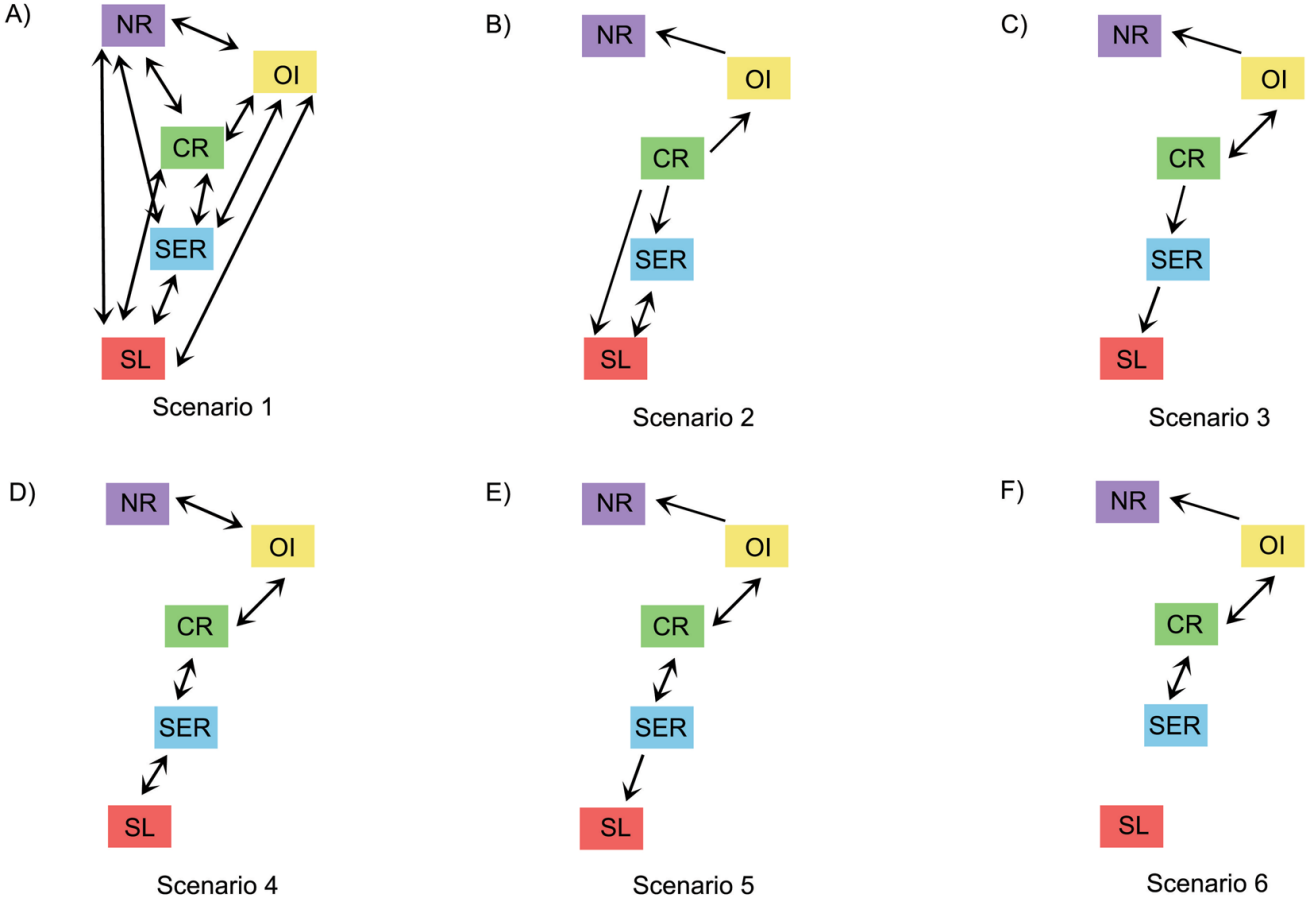
Scheme of migration patterns for all scenarios tested using Migrate. Arrows indicate the direction of gene flow considered in each scenario. Boxes and their colours correspond to populations of *Mussismilia hispida* as defined by Structure analysis, where NR: Northern Region, OI: Oceanic Islands, CR: Central Region, SER: Southeastern Region and SL: Southern Limit. Scenario 3 was the most probable one.

From the six migration scenarios tested (Fig. 3), the most probable one was scenario 3 (Fig. 3C), given that it had the highest values of both log marginal likelihoods and the highest probability among the ones tested (Table 4). In this scenario, Northern Region receives migrants from the Oceanic Islands, there is bidirectional gene flow between Oceanic Islands and Central Region, the Central Region exports migrants to the Southeastern Region and the latter exports migrants to the Southern Limit (Fig. 3C). The mutation-scaled effective population size (*Θ*) of the Northern Region was 0.008, the Oceanic Islands was 1.088, the Central Region was 1.988, the Southeastern Region was 0.188 and finally the Southern Limit was 0.008 (Supplementary Table S5). In this scenario, the number of migrants per generation is 12.32 from the Oceanic Islands to the Central Region and 7.61 from the Central Region back to the Oceanic Islands; 0.06 from the Oceanic Islands to the Northern Region; 0.49 from the Central Region to the Southeastern Region and 0.10 from the Southeastern Region to the Southern Limit (Supplementary Table S5).

**Table 4:** Log marginal Likelihood values for the six migration scenarios tested for *Mussismilia hispida* populations in Migrate-N.

Scenario	Bezier lmL	Harmonic lmL	Model probability
1	−2982632.34	−189324.33	0.000
2	−1407624.16	−257716.75	0.000
3	−1344425.35	−158866.12	1.000
4	−1483861.85	−193062.27	0.000
5	−1401576.81	−289244.22	0.000
6	−3030487.44	−793804.48	0.000

Values were calculated with Bezier approximation score (Bezier lmL) and Harmonic mean (Harmonic lmL). Model probability was calculated using Bezier marginal likelihoods.

## Discussion

The present study is the first to evaluate the genetic connectivity and migration patterns of *Mussismilia hispida*, one of the most important reef-building corals in the Southwestern Atlantic Ocean. These patterns were assessed across this species’ entire distributional range, encompassing more than 3,500 km of coast line, which is a great part of the Southwestern Atlantic (SWA) tropical region. Results show that *M*. *hispida*’s genetic structure can be explained by oceanographic processes, such as currents direction, upwelling events and past sea-level changes. Since gene flow intensity and direction and what barriers to dispersal may shape them are still unknown for most species in the region, we believe this study could serve as a baseline for hypothesis testing regarding the biogeographical barriers of sessile invertebrates in the SWA.

*M*. *hispida* individuals are structured into five populations according to Structure and F_ST_ analyses (North Region, Oceanic Islands, Central Region, Southeastern Region and Southern Limit), and the genetic diversity observed is similar to what has been found for other corals in the Atlantic with microsatellite data^8,9^. However, the two southernmost sites of *M*. *hispida*, had lower levels of genetic diversity. Reduced genetic diversity and higher genetic differentiation at peripheral populations appear to be common in animals and plants^20^ and has been observed for hydrocorals^21^ and scleractinian corals in the Southwestern Atlantic^13,15^, South Africa^6^ and Australia^22^. Interestingly, for *M*. *hispida* this pattern occurs in the southern range (Southeastern Region and Southern Limit), but it is not observed in the northern distributional edge (Northern Region). This asymmetry may be explained by the proximity of the Northern Region with the largest and more genetically diverse populations or to the stressful lower water temperatures^23,24^ in southernmost sites, that could limit dispersal or lead to selection of certain genotypes. All populations presented heterozygous deficits when compared with the expected under the Hardy-Weinberg Equilibrium (HWE), which appears to be common in microsatellite estimates of coral populations^6,25,26^. This deviation is likely due to assumptions of HWE not being met in natural populations. For instance, random mating is likely not met in coral populations, considering that adults are sessile and have high longevity, which can lead to generation overlap. Thus, inbreeding rates may be high for *M*. *hispida* and can be further reinforced by larval retention, but additional investigation is needed to confirm this.

Contradicting one of our initial hypotheses, the reproductive asynchrony reported for *M*. *hispida* does not appear to influence the observed patterns of gene flow. For example, in this study AB and PS individuals belong to the same population (Central Region) even though individuals in these two areas presented different spawning periods^16,17^. In contrast, individuals from Armação dos Búzios (BZ) and Santos have similar reproduction periods^18,19^, while genetic analyses placed individuals from BZ and IB (90 km north of Santos) in different populations (Southeastern Region vs. Southern Limit). Asynchrony in spawning times has been observed for other corals, like *Pocillopora damicornis*, and it was explained as either the result of local colonization by different populations with different spawning times or the existence of cryptic species^27^. For *M*. *hispida*, the presence of another species is unlikely given the high genetic similarity found among individuals in AB and PS using hyper-variable markers. However, it is possible that differences in spawning time of AB and PS are due to different local abiotic conditions or cues to spawning.

The estimated migration rates and observed genetic diversity demonstrate how is the source-sink dynamics for *M*. *hispida*. Oceanic Islands and Central Region populations can be considered sources of migrants to the other ones. Such pattern is expected for the Central Region considering the central-margin hypothesis^20^, given that this population covers approximately 1,600 km of coastline, includes the largest coral reef system on the SWA (the Abrolhos Reef Complex) and has the highest mutation-scaled effective population size. Conversely, the high genetic diversity and migration patterns for the Oceanic Islands is highly unexpected, given that islands usually are considered peripheral and a low genetic diversity was expected^20^. Finally, the Northern Region and Southern Limit can be considered sink populations, as expected for populations at the limit of a species’ distribution^20^.

Connectivity and migration patterns of the Northern Region can be explained by the main ocean currents in the area. The South Equatorial Current (SEC) that crosses the Atlantic Ocean at 5–10°S splits into two major oceanic currents in the SWA that follow the coast of Brazil: the North Brazil Current (NBC), that flows in a northwestern direction, and the Brazil Current, a western boundary current that flows southwards (Fig. 1)^28,29^. The northwestern flow of the NBC could explain why there is genetic structure between the Northern Region and the remaining populations and the migration from Oceanic Islands to the Northern Region. A genetic break in this area also appears to be shared by the corals *Favia gravida* and *Siderastrea radians*^13^, but this is not general for all invertebrates^30^. Besides, the separation of the Northern Region population found here does not match previous biogeographic studies, such as the subdivisions of coastal marine provinces in the tropical Atlantic^31^ and reef fish biogeography^32^. The NBC current may not limit gene flow from most species, but its role as a barrier to dispersal appears to be important for corals.

The Central Region population had high levels of connectivity, possibly because this region is under the homogenizing influence of the Brazil Current. Although no structure was found for *M*. *hispida* in this region, possible barriers to dispersal have been reported, such as the São Francisco River mouth (10°30′S)^33^, the entrance to the Todos os Santos Bay and the adjacent Salvador canyon^34^. Spalding *et al*.^31^ considered this whole area as a single marine province (Tropical Southwestern Atlantic), including the oceanic islands, and recognize a possible subdivision of ecoregions nearby Salvador (13° S). For the scleractinians *F*. *gravida* and *S*. *radians*, a genetic discontinuity has been observed between JP and AB locations^13^, which agrees with Spalding *et al*.^31^. This could be explained by a more limited dispersal and differences in reproductive modes, since *M*. *hispida* is a broadcast spawner and *F*. *gravida* and *S*. *radians* are brooders^17,35^, although reproductive mode not always explains differences in connectivity patterns^25^. Besides, this area has also been shown to limit the distribution of two *Millepora* species^21^ and the diversity of *Symbiodinium* associated with *M*. *hispida*^36^. However, it is important to note that there is few information on PLD and coral coverage for SWA corals and the influence of these factors shaping the apparent distinct connectivity of these species can only be speculated. Nevertheless, these potential barriers do not appear to be effective for *M*. *hispida* and the occurrence of barriers to dispersal between 10–13°S in the SWA may not be generalized for all marine organisms.

The break separating the Central Region from the Southeastern Region in *M*. *hispida* is also shared with the coral *S*. *stellata*^14^ and reef fish, including genetic^37,38^ and biogeographic studies^32^.This particular area (18–23°S) has been considered a transitional zone almost since the beginning of zoogeographical studies^31,32,39^ and is characterized for having upwellings and higher prevalence of colder water masses^23,24,29^. However, since the migration pattern estimated here shows gene flow from the Central to the Southeastern Region, such oceanographic processes may act more as a filter than as a barrier in this specific area. Furthermore, there is another genetic break for *M*. *hispida* near this colder water region, separating the Southeastern Region and the Southern Limit. This could be explained by the strongest upwelling events along the Brazilian coast, located in capes São Tomé and Frio (21–23°S)^24,40^. In fact, zooxanthellate coral diversity is greatly reduced south of this region, with only two species occurring down to São Paulo State (*M*. *hispida* and *Madracis decactis*) and only one species occurring as far south as Santa Catarina State (*M*. *decactis*)^11^. Upwelling events have been shown to limit the settlement of invertebrate larvae^41,42^ and this may be the case for other species and for *M*. *hispida*. Interestingly, the rocky shores immediately south of Cape Frio, which are under the strongest influence of the upwelling, have a distinct fauna with subtropical characteristics, where no scleractinian corals are found^43,44^. On the other hand, the more protected area north of this headland has a tropical fauna that includes zooxanthellate scleractinians^11^ and corresponds to the sites AC and BZ from the present study. However, this protected area also has cold water temperatures, and temperature may not be the only factor that prevents coral recruitment in the area, which could also be influenced by local hydrodynamism.

The upwellings in São Tomé and Frio’s capes could act as a barrier to *M*. *hispida* in two ways: 1) larvae may rarely cross into or out of the cold-water region because currents or temperature may impede dispersal or 2) local selection limits recruitment of certain genotypes in the region. Upwelling in the Cape Frio typically occurs during the austral spring and summer^45^, coinciding with *M*. *hispida* spawning in the region^18^. Since sea water temperatures during these events can drop to 11 °C^45^, larvae formation could be inhibited, as shown for the coral *Oculina varricosa* with temperatures ranging from 10–17 °C^46^. Upwellings can also create retention zones due to water stratification^47^, which would hinder larval dispersal. Selection may also play a role, as local environmental conditions could favour the survival of certain genotypes adapted to lower temperatures or other characteristics of the area. Furthermore, the processes causing reduced dispersal and selective pressure may act synergistically to restrict connectivity. While the exact way these upwellings act as a barrier could not be discerned, the present data indicate that they restrict gene flow for *M*. *hispida* and could possibly do so for other marine organisms. Indeed, the Cape Frio upwelling has been suggested as a biogeographic barrier based on levels of endemicity^48^ and many marine organisms in the SWA appear to have their southern distribution limit near this area, including several coral species^11^. However, to our knowledge, these are the first genetic data to support its role as a barrier for a marine invertebrate.

The patterns of genetic diversity, connectivity and migration concerning the Oceanic Islands is one exception that has no obvious explanation in the currents flow or prevailing water masses. Four unexpected patterns were observed: the high gene flow between Trindade Island (TR) and the other two oceanic islands; the restricted gene flow of these sites with their geographically closer mainland; the high genetic diversity of the Oceanic Islands; and the apparent exchange of propagules between Oceanic Islands and Central Region populations. The high gene flow among the three Oceanic Islands sites was not expected due to: i) the presence of a large oceanic current (NBC) that runs westwards crossing Fernando de Noronha archipelago (FN) and Rocas Atoll (AR), separating these two from TR, which lies far to the south; ii) the large distance that separates TR from both FN and AR (~1,800 km); and iii) the lack of nearby shallow reef habitats between TR and FN-AR, which would increase this species dispersal capabilities^22^. Since the direction of the SEC (that originates NBC) varies seasonally^49^ and larvae could potentially reach the edge of the continental shelf depending on the period, this current may not prevent gene flow among these sites. Nevertheless, a high dispersal capability and/or intermediate habitats would be needed to maintain this gene flow. Long distance connectivity has been observed in other corals, but this is usually observed in species with a long PLD, such as *Acropora millepora* (200 days)^7^, which is not the case of *M*. *hispida* (PLD≈10 days)^17^. Ocean currents patterns could also explain the high dispersal capability, but these are difficult to estimate given that they vary with latitude, season and proximity of the coast, and an oceanographic modelling approach would be needed to understand the influence of these processes. Other fact that could also account for this pattern is the occurrence of *M*. *hispida* in mesophotic reefs, especially in seamounts and/or wave-cut erosion steps and beachrocks in the continental shelf margin^50^. Such mesophotic populations could provide a more continuous patch of suitable habitats, favouring the maintenance of gene flow between the Oceanic Islands. Complex currents coupled with populations in intermediary mesophotic reefs may account for this unexpected observed connectivity.

The second unexpected pattern is the genetic break between the Oceanic Islands’ sites and the mainland. Such differentiation has also been observed for the shark *Ginglymostoma cirratum*^51^ and the queen angelfish *Holacanthus ciliaris*^38^. This region is also considered a biogeographic break for reef fish^32^ and distinct ecoregions in the tropical Atlantic^31^. This could be due to differences in coastal vs. open ocean environments and currents pattern. The Brazil Current may maintain offshore *M*. *hispida* localities well mixed, while closer to the coast different factors limit offshore dispersal, such as river outflows, high turbidity, and complex coastal circulation patterns. This would explain the separation of the islands and the geographically closer mainland sites. Still, that was not expected for TR, since it is connected to the mainland via the Vitoria-Trindade seamount chain^52^, which acts as stepping-stone for reef fish species^53^. However, genetic connectivity between TR and the mainland is not observed, even though *M*. *hispida* colonies have been recorded in mesophotic reefs along this seamount chain and on the continental shelf north of TR^54^. One explanation is that TR may be isolated from the mainland due to deep water channels that exist between the island itself, the Columbia Seamount, and the Dogaressa Bank^52^. Currents along these channels could restrict coral larvae dispersal, which is more limited in comparison to fish^55^. In general, few genetic studies have been conducted in the SWA that include both the mainland and oceanic islands and what causes this probable barrier to dispersal is an interesting feature that warrants further investigation.

The third unexpected pattern is the high genetic diversity of Oceanic Islands population. Under the perspective of island biogeography, the oceanic islands are not expected to harbour large genetic diversity^56^. This is because all three islands have relatively small areas, two of them (AR and FN) are separated from the mainland with no intermediary suitable habitats and *M*. *hispida* appears to have low densities in all of them (LP and CZ, personal observation). With this, one would expect that the extinction risk in these areas is high and their population is mainly dependent on subsequent colonization. Nonetheless, this scenario would unlikely result on the formation of a distinct genotypic cluster with high genetic diversity, like the one observed here. One possible explanation is that the oceanic islands have been isolated from the mainland sites long enough to accumulate the observed levels of genetic diversity, however this goes against the fourth unexpected pattern found here, the mutual exchange of migrants between the Oceanic Islands and Central Region. Therefore, an alternative explanation may account for both patterns.

This mutual exchange of migrants is not obviously related to oceanographic currents, but there are complex vortices of the Brazil Current near southern Bahia and Espírito Santo^57,58^ (Fig. 1) that could facilitate dispersal through mixing of water masses. The addition of genetic information from unsampled mesophotic sites between these two populations may help explain how migrants from the mainland make their way back to the islands. However, an alternative explanation is that the oceanic islands acted as refugia during a period of lower sea level stance, which would also explain the high genetic diversity of these sites. During the Last Glacial Maximum ca. 30,000-19,000 years ago, sea level was approximately 130 m lower than the present-day and at least 40 m lower 10,000 years ago^59–61^. At this time, *M*. *hispida* from shallow water coastal environments along the mainland disappeared, while the oceanic islands, seamounts and the continental margin could have their slopes colonized. As sea level increased over the late Pleistocene and Holocene, coastal environments would have to be subsequently recolonized, potentially from offshore sites, including the Oceanic Islands. The coastal Brazilian reefs are estimated to be around 7,000 years old^62^, which postdates the period of low sea-level stance, being indicative of coastal recolonization at more recent times. In fact, seamounts acting as refugia in the SWA has already been hypothesised in previous studies for scleractinian corals^63^ and reef fish^53^. In this scenario, this refugia populations survived the extinction caused by sea-level changes and maintained the genetic diversity that was lost in coastal areas, similarly to what was proposed for paleoendemic reef fish species in the Vitória-Trindade seamount chain^53^. Furthermore, the oceanic islands acting as refugia in the past, harbouring high genetic diversity and exchanging migrants with the Central Region population is consistent with the biodiversity feedback model^64^, where peripheral habitats can also export diversity.

Preserving current gene flow among natural populations is necessary to maintain evolutionary processes and, therefore, is essential for a proper long term management^2^. Likewise, isolated populations need attention in terms of management, so that their continuity can be assured. Our results suggest that Southeastern Region and Southern Limit populations are isolated from one another and from more central populations, despite their geographic proximity. This highlights the need for management and the establishment of new marine reserves within each of these genetic populations. The Oceanic Islands and Central Regions were identified as the main source of genetic diversity and migrants and effective management measures need to be enforced and evaluated to ensure that coral populations continue to thrive in these regions. Therefore, the understanding of *M*. *hispida* population limits presented here is of great importance and should be taken into consideration in future conservation planning for Brazil’s marine habitats.

## Methods

### Sample collection

Fifteen sites were sampled along the Brazilian coast (Fig. 1), covering the entire distributional range of *Mussismilia hispida*, with twelve continental sites: Parcel do Manuel Luís (PML), Maranhão State; Fortaleza (FZ), Ceará State; João Pessoa (JP), Paraíba State; Tamandaré (TE), Pernambuco State; Salvador (SA), Porto Seguro (PS), and Abrolhos Reef Complex (AB), Bahia State; Guarapari (GP), Espírito Santo State; Armação dos Búzios (BZ), Arraial do Cabo (AC), and Ilha Grande (IG), Rio de Janeiro State; Ilhabela (IB), São Paulo State. Additionally, three oceanic sites were sampled: Rocas Atoll (AR), Rio Grande do Norte State; Fernando de Noronha (FN), Pernambuco State; and Trindade Island (TR), Espírito Santo State (Fig. 1). Fragments of ~0,5 cm^2^ were obtained using a hammer and chisel. Between seven to 40 colonies were sampled per site depending on local abundance, totalling 391 samples (Table 1). All samples were directly stored in a CHAOS lysis buffer^65^ (4 M Guanidine Thiocyanate, 0.5% n-Lauroylsarcosine Sodium, 25 mMTris–HCl pH 8.0, 0.1 M B-mercaptoethanol) until DNA extraction.

### DNA extraction and genotyping

Genomic DNA was extracted using phenol:chlorophorm according to Fukami *et al*.^65^. DNA quality and concentration were assessed with the pattern Lambda DNA (125 ng/µL) on a 0.8% agarose gel stained with GelRed (Biotium) and visualized under UV light.

Thirteen species-specific microsatellite *loci* (Mhi1, Mhi2, Mhi4, Mhi14, Mhi16, Mhi17, Mhi18, Mhi20, Mhi21, Mhi23, Mhi24, Mhi26, Mhi27)^66^ were amplified by PCR, following Schuelke’s protocol of tailed primers^67^. Each PCR contained 1U GoTaq (Promega), 5 × PCR Buffer (Promega), 200 µM dNTP mix (Invitrogen), 1.5–2.5 mM MgCl_2_, 1 mg/ml BSA (Invitrogen), 0.2 μM of forward tailed primer, 0.4 μM of fluorochrome labeled primer, and 0.8 μM of reverse primer in 10 μL reactions with approximately 5–10 ng of DNA template. Cycling conditions had an initial cycle at 95 °C for 3 min; followed by 5 cycles at 95 °C for 30 s, 52–62 °C for 30 s, 72 °C for 45 s; with an additional 35 cycles at 92 °C for 30 s, 52–62 °C for 30 s, 72 °C for 55 s; and a final cycle at 72 °C for 30 min. Concentration of MgCl_2_ and annealing temperatures varied according to the marker and followed Zilberberg *et al*.^66^. *Loci* amplification success, overall size and concentration were assessed using a 100 bp DNA ladder (Fermentas) on a 2% agarose gel stained with GelRed (Biotium) and visualized under UV light.

Up to four PCR products with different fluorescent dyes were pooled together and were genotyped in an ABI3500 sequencer using a GS600-LIZ size standard (Applied Biosystems). Allele sizes were scored manually using the software GeneMarker (Soft Genetics). At each run, two to three samples that had previously been genotyped were re-genotyped as a positive control and to ensure that allele scores were consistent. Samples with poor genotype resolution in a *locus* were re-amplified and re-genotyped once. If poor resolution was observed twice, that *locus* was left blank for the particular sample. Only samples with more than nine genotyped *loci* were used in the analyses.

### Data analyses

All *loci* were tested for null alleles using Micro-Checker^68^ and linkage disequilibrium using FSTAT^69^. *Loci* that presented probability of null alleles for the majority of locations or linkage disequilibrium were removed from the subsequent analyses. The number of alleles per *locus*, the number of exclusive alleles per locality and the observed and expected heterozygosity for each site were calculated using the Microsatellite toolkit^70^. *Loci* statistics were calculated using FSTAT. F_IS_ indexes were calculated to evaluate deviations from Hardy-Weinberg equilibrium using Genetix^71^, assuming sampling sites (localities) as populations.

Population structure was assessed using pairwise F_ST_ fixation indexes, assuming localities as populations, with 1,000 permutations, in Genetix. Principal coordinates analysis (PCoA) of Fst values among localities was also calculated using GenAlEx^72^. The existence of isolation by distance was verified with a Mantel Test performed in IBDWS^73^. F_ST_ values were used for genetic distances and pairwise geographic distances in kilometres were measured in Google Earth (http://maps.google.com/) as straight paths between localities that did not cross the continent. Additionally, a Bayesian clustering algorithm was implemented using the software Structure 2.3.4^74^ without *a priori* information of sampling location. The analysis was made with an admixture ancestry model and correlated allelic frequencies. Each analysis had 1,000,000 Markov-Monte Carlo chains from which 200,000 were discarded (burnin chains). The number of probable populations (*K*) tested was from 1 to 15, with ten iterations each. The best value of *K* was defined using the higher likelihood mean (LnPD)^74^ as suggested by Waples and Gaggiotti^75^ and the results were visualized using Structure Harvester^76^. A second analysis was made with the same parameters but using sampling locations as priors. One bar plot summarizing each value of *K* was generated using CLUMPP^77^ and Distruct^78^. Furthermore, each previously defined population was analysed individually in Structure with the same parameters as the first analysis to verify the existence of substructure.

Migration rates and different migrations scenarios were estimated using Migrate-N^79^. Genetic clusters determined by Structure were used as populations, given the limited sample size in some localities, the low genetic differentiation of localities from the same cluster and the substantial increase in model parameters if more populations were tested. The Brownian model for microsatellite data was used with constant migration rates. All analyses were made by Bayesian inference with constant distribution for *Θ* and *M* priors from 0.0 to 20.0 and 0.0 to 200.0, respectively. Four long chains were run with static heating between them, where there were 40,000 recorded steps and 10,000 steps of burnin for each chain. The mutation-scaled effective population size *Θ* was estimated for each population, where ${\Theta }_{i}=4\,x\,Ne\,x\,\mu$, and *μ* is the mutation rate per site per generation, calculated based on the microsatellite data by the program. The number of migrants per generation (Nem) between localities was calculated using the values of *Θ* and *M* estimated in each run of Migrate, where $Ne{m}_{i-j}={\Theta }_{j}\times {M}_{i-j}$ . Six migration scenarios (Fig. 3) were tested to evaluate which one best explained the observed patterns of gene flow among populations, as proposed by Beerli and Palczewski^80^. The tested scenarios were chosen based on known biogeographic breaks^31,32^, on the results of the population structure analyses and on the main oceanic currents flow in the Southwestern Atlantic (SWA). The first scenario simulated panmixia, where all possible gene flow directions were accounted for (Fig. 3A). The second scenario simulated gene flow between the populations that had the highest observed posterior distribution values of migration (mode > 20,000) in Scenario 1(Fig. 3B), while in the third scenario, gene flow was set to simulate the direction of the main oceanic currents in the SWA, but with bidirectional gene flow between the populations that covered the largest areas (Fig. 3C). The fourth scenario allowed bidirectional gene flow between all neighbouring populations, following a stepping stone migration model (Fig. 3D). The fifth scenario also simulated the direction of the main currents, but with bidirectional gene flow between the central population and its neighbours (Fig. 3E). Finally, the sixth scenario was similar to scenario 5, but in this one the southernmost population is isolated from the others (Fig. 3F). The best scenario was chosen based on the higher Bezier and Harmonic Mean log marginal likelihoods and on the model probability, calculated using Bezier marginal likelihoods in Wolfram Mathematica Software, where ${\rm{Prob}}({{\rm{model}}}_{i})=\,\frac{m{{\rm{L}}}_{{{\rm{model}}}_{i}}}{{\sum }_{j}^{i}m{{\rm{L}}}_{{{\rm{model}}}_{j}}}$. Although we believe that the scenarios tested are the most realistic ones given the previous information available, it is important to note the limitation of analysing only a small portion of all possible migration scenarios and that the stipulated best scenario is relative to this specific set of scenarios.

Input files for all analyses except the one made with the Microsatellite toolkit were generated using CREATE software^81^.

### Data availability

The datasets analysed during the current study are available from the corresponding author on reasonable request.

## Electronic supplementary material

Supplementary material
