# Supplementary material for: Contemporary and historical oceanographic processes explain genetic connectivity in a Southwestern Atlantic coral
Source: Sci Rep. 2018 Feb 8;8:2684. doi: 10.1038/s41598-018-21010-y (PMC5805724; doi:10.1038/s41598-018-21010-y)

Supplementary material

Contemporary and historical oceanographic processes explain genetic connectivity in Southwestern Atlantic corals

L. Peluso, V. Tascheri, F. Nunes, C. B. Castro, D. O. Pires, C. Zilberberg

Table of contents

Table S1	Page 2
Table S2	Page 2
Table S3	Page 3
Table S4	Page 4
Table S5	Page 5
Figure S1	Page 6
Figure S2	Page 7
Figure S3	Page 7

Table S1: Allelic richness per locus per sampling site. Calculations were based on 10 diploid individuals. PML was excluded due to low sample size (analysis would be based only in 4 diploid individuals).

	FZ	AR	FN	JP	TE	SA	PS	AB	GP	TR	BZ	AC	IG	IB
Mhi1	9.238	9.504	8.635	8.022	6.270	6.007	6.113	7.636	5.643	9.924	4.944	5.854	1.886	1.761
Mhi2	8.470	9.929	10.602	12.009	8.655	9.427	9.787	9.915	9.000	10.134	7.457	8.711	4.827	4.598
Mhi14	7.818	5.555	6.162	5.000	6.419	6.556	5.951	5.275	4.812	7.442	4.618	3.909	2.999	4.078
Mhi16	1.575	2.430	2.773	4.456	2.426	5.116	2.124	3.042	1.996	1.978	1.867	1.945	3.807	2.635
Mhi17	3.479	2.000	2.499	1.986	3.564	4.299	3.215	3.276	3.810	3.225	1.000	1.000	1.993	1.985
Mhi18	7.675	9.243	8.982	7.603	9.676	7.938	8.655	7.363	9.933	10.358	8.173	6.773	6.200	5.106
Mhi20	10.961	6.300	6.286	9.936	10.152	11.727	8.830	10.138	9.411	9.683	6.882	7.855	2.602	4.385
Mhi21	8.116	10.706	10.914	8.053	9.076	6.004	7.452	7.154	7.889	7.984	5.536	4.916	3.525	3.905
Mhi23	6.097	7.015	7.422	6.341	8.414	9.960	9.463	11.397	3.976	7.263	4.219	4.511	2.706	3.160
Mhi26	7.891	7.609	8.581	8.274	6.846	7.210	7.589	8.505	9.143	8.572	6.493	6.837	5.948	6.436

Table S2: Allelic richness per locus per population, as defined by Structure. Calculations were based on 28 diploid individuals.

	NR	OI	CR	SER	SL
Mhi1	13.997	16.146	10.803	8.567	1.996
Mhi2	16.618	18.766	16.481	10.975	6.005
Mhi14	9.536	8.864	8.405	4.752	4.406
Mhi16	2.906	4.252	6.176	2.000	5.087
Mhi17	5.462	3.692	4.450	1.000	2.000
Mhi18	13.895	20.350	15.410	11.035	8.020
Mhi20	18.040	13.344	17.358	12.286	5.576
Mhi21	10.712	17.660	10.637	7.727	5.860
Mhi23	9.000	11.827	14.730	5.848	4.159
Mhi26	12.918	13.644	11.340	7.948	7.688

Table S3: Characterization of the ten microsatellite *loci* used in *Mussismilia hispida* for each site sampled. The number of alleles (A), observed heterozygosity (Ho), expected heterozygosity (He) and F_{IS} calculated for each locus for each location (F_{IS}) are given. Bold numbers of F_{IS} indicate adjusted significant values (p<0.0003). Sampling sites are abbreviated as in Table 1.

	PML	FZ	AR	FN	JP	TE	SA	PS	AB	GP	TR	BZ	AC	IB	IG
Mhi1															
A	8	12	13	16	10	8	8	11	13	6	15	7	9	2	2
Ho	0.714	0.793	0.579	0.650	0.688	0.353	0.571	0.485	0.485	0.750	0.720	0.346	0.613	0.128	0.059
He	0.901	0.886	0.898	0.859	0.843	0.733	0.648	0.584	0.748	0.710	0.895	0.603	0.770	0.166	0.112
F_{IS}	0.221	0.106	**0.361**	**0.246**	0.189	**0.526**	0.121	0.172	**0.355**	-0.059	0.199	0.430	0.207	0.228	0.480
Mhi2															
A	9	14	14	21	15	10	12	15	17	9	17	10	11	7	6
Ho	0.714	0.759	0.684	0.795	0.875	0.824	0.727	0.727	0.882	0.700	0.654	0.792	0.806	0.526	0.545
He	0.879	0.858	0.892	0.895	0.940	0.863	0.901	0.892	0.897	0.889	0.865	0.854	0.887	0.609	0.660
F_{IS}	0.200	0.117	0.238	0.114	0.071	0.047	0.196	0.187	0.016	0.222	0.248	0.074	0.092	0.138	0.176
Mhi14															
A	5	10	6	9	6	8	8	8	7	5	11	5	4	3	6
Ho	0.429	0.966	0.895	0.875	0.533	0.588	0.818	0.727	0.471	0.583	0.767	0.600	0.258	0.500	0.471
He	0.725	0.860	0.795	0.782	0.722	0.766	0.800	0.752	0.712	0.743	0.822	0.744	0.694	0.670	0.665
F_{IS}	0.429	-0.126	-0.129	-0.120	0.268	0.238	-0.023	0.033	0.342	0.222	0.068	0.196	**0.632**	0.256	0.296
Mhi16															
A	2	2	3	6	6	3	6	4	6	2	3	2	2	6	4
Ho	0.000	0.069	0.105	0.200	0.313	0.059	0.318	0.121	0.176	0.182	0.111	0.077	0.161	0.538	0.235
He	0.264	0.068	0.198	0.190	0.389	0.169	0.737	0.118	0.221	0.173	0.108	0.145	0.204	0.446	0.218
F_{IS}	1.000	-0.018	0.474	-0.054	0.202	0.660	**0.574**	-0.028	0.205	-0.053	-0.026	0.474	0.211	-0.210	-0.080
Mhi17															
A	3	5	2	4	2	4	5	4	4	4	5	1	1	2	2
Ho	0.143	0.414	0.526	0.425	0.250	0.529	0.591	0.455	0.324	0.167	0.100	0.000	0.000	0.385	0.324
He	0.385	0.405	0.501	0.428	0.226	0.512	0.689	0.443	0.377	0.431	0.462	0.000	0.000	0.315	0.275
F_{IS}	0.647	-0.023	-0.053	0.008	-0.111	-0.036	0.146	-0.026	0.143	0.624	**0.786**	NA	NA	-0.226	-0.179
Mhi18															
A	6	15	14	19	10	12	12	17	13	11	22	12	9	12	6
Ho	0.429	0.724	0.526	0.316	0.438	0.563	0.682	0.758	0.647	0.750	0.733	0.846	0.774	0.846	0.853
He	0.681	0.776	0.747	0.741	0.786	0.895	0.828	0.827	0.701	0.848	0.804	0.849	0.802	0.792	0.773
F_{IS}	0.390	0.067	0.301	0.577	**0.452**	0.379	0.180	0.085	0.079	0.120	0.089	0.004	0.035	-0.069	-0.106
Mhi20															
A	9	18	8	10	12	12	17	15	17	10	16	10	13	4	6
Ho	0.857	0.846	0.737	0.625	0.875	0.706	0.762	0.813	0.735	0.750	0.643	0.654	0.733	0.205	0.529
He	0.934	0.912	0.691	0.669	0.905	0.913	0.919	0.843	0.889	0.899	0.875	0.771	0.827	0.193	0.582
F_{IS}	0.089	0.073	-0.068	0.067	0.034	0.232	0.174	0.036	0.175	0.172	**0.269**	0.154	0.115	-0.063	0.092
Mhi21															
A	5	10	14	21	10	11	7	10	10	8	11	8	7	5	6
Ho	0.286	0.414	0.563	0.632	0.563	0.500	0.400	0.452	0.500	0.667	0.793	0.542	0.552	0.242	0.294
He	0.813	0.855	0.879	0.895	0.784	0.847	0.815	0.837	0.837	0.880	0.819	0.680	0.686	0.369	0.424
F_{IS}	**0.667**	**0.521**	0.368	**0.297**	0.289	**0.417**	**0.516**	**0.464**	**0.406**	0.251	0.032	0.207	0.199	0.346	0.310
Mhi23															
A	4	8	8	12	7	10	14	15	18	4	10	5	7	4	4
Ho	1.000	0.958	0.867	0.765	0.250	0.563	0.773	0.636	0.618	0.500	0.800	0.458	0.484	0.256	0.324
He	0.786	0.732	0.805	0.817	0.817	0.845	0.872	0.896	0.921	0.685	0.799	0.700	0.714	0.543	0.545
F_{IS}	-0.333	-0.318	-0.080	0.064	**0.701**	0.341	0.116	**0.293**	**0.333**	0.279	-0.002	0.350	0.326	**0.531**	0.410
Mhi26															
A	9	12	22	15	10	8	9	11	13	10	14	7	9	8	9
Ho	1.000	0.828	0.684	0.838	0.688	0.923	0.591	0.781	0.848	0.833	0.633	0.577	0.677	0.744	0.794
He	0.939	0.856	0.708	0.835	0.867	0.797	0.835	0.852	0.839	0.891	0.821	0.817	0.844	0.795	0.811
F_{IS}	-0.071	0.034	0.035	-0.003	0.212	-0.166	0.297	0.084	-0.012	0.068	0.232	0.298	0.200	0.065	0.021
Overall															
Ho	0.557	0.677	0.617	0.612	0.547	0.561	0.623	0.595	0.569	0.588	0.595	0.489	0.506	0.437	0.443
He	0.731	0.721	0.711	0.711	0.728	0.734	0.804	0.704	0.714	0.715	0.727	0.616	0.643	0.490	0.507
F_{IS}	**0.250**	0.062	0.137	**0.141**	**0.255**	**0.242**	**0.229**	**0.157**	**0.206**	**0.184**	**0.184**	**0.209**	**0.216**	0.109	**0.128**

Table S4: Characterization of the ten microsatellite *loci* used in *Mussismilia hispida* for each population as defined by Structure. The number of alleles (A), observed heterozygosity (Ho), expected heterozygosity (He) and F_{IS} calculated for each locus for each population (F_{IS}) are given. Bold numbers of F_{IS} indicate adjusted significant values (p<0.001).

		Norhtern Region	Oceanic Islands	Central Region	Southeastern Region	Southern Limit
Mhi1						
	A	15	22	20	10	2
	Ho	0.778	0.655	0.530	0.491	0.096
	He	0.888	0.893	0.703	0.717	0.140
	F_{IS}	0.022	**0.001**	**0.001**	**0.001**	0.047
Mhi2	N					
	A	19	30	29	12	7
	Ho	0.750	0.726	0.795	0.800	0.535
	He	0.879	0.909	0.908	0.872	0.633
	F_{IS}	0.021	**0.001**	**0.001**	0.096	0.024
Mhi14						
	A	10	11	10	5	6
	Ho	0.861	0.843	0.624	0.411	0.486
	He	0.853	0.817	0.755	0.711	0.669
	F_{IS}	0.642	0.803	**0.001**	**0.001**	**0.001**
Mhi16						
	A	3	7	7	2	6
	Ho	0.056	0.151	0.188	0.123	0.397
	He	0.108	0.166	0.345	0.176	0.349
	F_{IS}	0.022	0.201	**0.001**	0.078	1.000
Mhi17						
	A	6	6	2	1	2
	Ho	0.361	0.337	0.403	0.000	0.356
	He	0.397	0.458	0.461	0.000	0.295
	F_{IS}	0.300	0.004	0.030		1.000
Mhi18_d						
	A	16	39	30	14	12
	Ho	0.667	0.506	0.654	0.807	0.849
	He	0.763	0.764	0.817	0.821	0.781
	F_{IS}	0.082	**0.001**	**0.001**	0.448	0.953
Mhi20						
	A	19	19	27	16	7
	Ho	0.848	0.655	0.773	0.696	0.356
	He	0.925	0.764	0.894	0.802	0.400
	F_{IS}	0.077	0.005	**0.001**	0.019	0.104
Mhi21						
	A	11	27	16	9	8
	Ho	0.389	0.675	0.496	0.547	0.269
	He	0.852	0.893	0.847	0.685	0.398
	F_{IS}	**0.001**	**0.001**	**0.001**	0.012	**0.001**
Mhi23						
	A	9	13	19	7	5
	Ho	0.964	0.797	0.586	0.473	0.288
	He	0.734	0.803	0.872	0.707	0.551
	F_{IS}	1.000	0.504	**0.001**	**0.001**	**0.001**
Mhi26						
	A	14	17	17	9	9
	Ho	0.857	0.733	0.773	0.632	0.767
	He	0.874	0.812	0.847	0.833	0.802
	F_{IS}	0.474	0.019	0.011	**0.001**	0.263
Overall						
	Ho	0.653	0.608	0.582	0.498	0.440
	He	0.727	0.728	0.745	0.632	0.502
	F_{IS}	**0.103**	**0.166**	**0.219**	**0.214**	**0.124**

Table S5**:** Values of the mutation-scaled effective population size (*Θ*) and the number of migrants per generation (Nem) of *Mussismilia hispida* for all six migration scenarios tested on Migrate, where NR: Northern Region, OI: Oceanic Islands, CR: Central Region, SER: Southeastern Region and SL: Southern Limit.

	Scenario 1	Scenario 2	Scenario 3	Scenario 4	Scenario 5	Scenario 6
Θ_{NR}	0.263	0.038	0.008	0.008	0.008	0.143
Θ_{OI}	0.473	1.463	1.088	0.803	1.358	0.008
Θ_{CR}	1.493	0.008	1.988	1.178	0.383	0.008
Θ_{SER}	0.263	0.053	0.188	0.308	0.008	0.248
Θ_{SL}	0.233	0.143	0.008	0.218	0.473	0.233
Nem NR>OI	5.95	-	-	4.65	-	-
Nem NR>CR	5.67	-	-	-	-	-
Nem NR>SER	5.09	-	-	-	-	-
Nem NR>SL	1.91	-	-	-	-	-
Nem OI>NR	5.62	0.16	0.06	0.09	0.04	1.17
Nem OI>CR	25.97	-	12.32	7.30	2.07	0.06
Nem OI>SER	4.25	-	-	-	-	-
Nem OI>SL	3.30	-	-	-	-	-
Nem CR>NR	3.62	-	-	-	-	-
Nem CR>OI	11.25	2.63	7.61	3.05	3.53	0.03
Nem CR>SER	7.72	0.24	0.49	1.78	0.02	1.34
Nem CR>SL	7.11	0.26	-	-	-	-
Nem SER>NR	4.88	-	-	-	-	-
Nem SER>OI	5.20	-	-	-	-	-
Nem SER>CR	18.81	-	-	4.95	-	0.03
Nem SER>SL	7.86	2.14	0.10	4.48	6.14	-
Nem SL>NR	1.94	-	-	-	-	-
Nem SL>OI	8.03	-	-	-	-	-
Nem SL>CR	27.16	-	-	-	-	-
Nem SL>SER	6.67	0.58	-	15.19	-	-

Figure S1: Values of LnPD (A) and Delta K (B) for the *K* values tested in the Structure analysis, ranging from 1 to 15. The best value of K was defined using the higher likelihood mean (LnPD), as suggested by Waples and Gaggiotti (2006).

A)

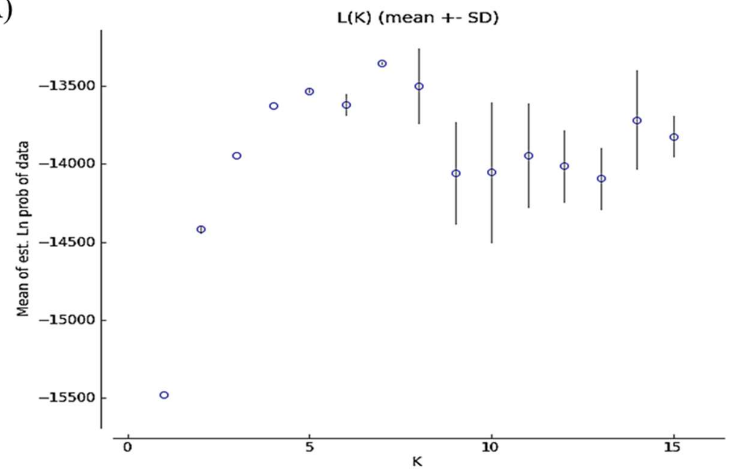

B)

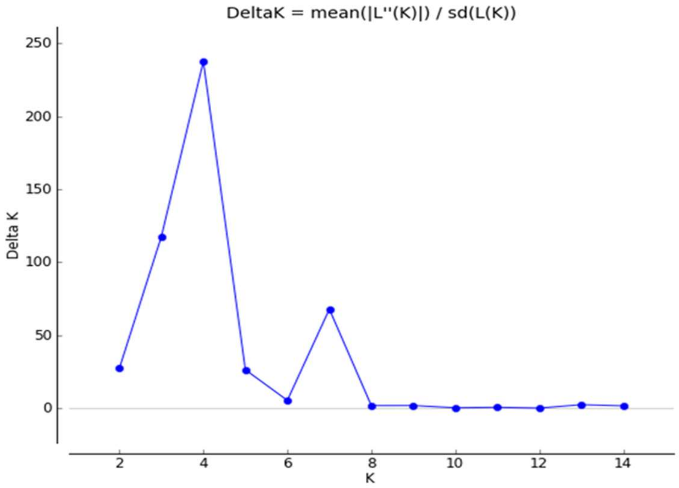

Figure S2: Bar plot showing the average probability of membership of each sample (vertical bars) in each population (colors) for *K*=1 to *K*=7 of *Mussismilia hispida*. Analyses were made with 10 iterations and with sampling locations as prior. Sampling sites are in the y-axis separated by vertical black lines, abbreviated as in Table 1.

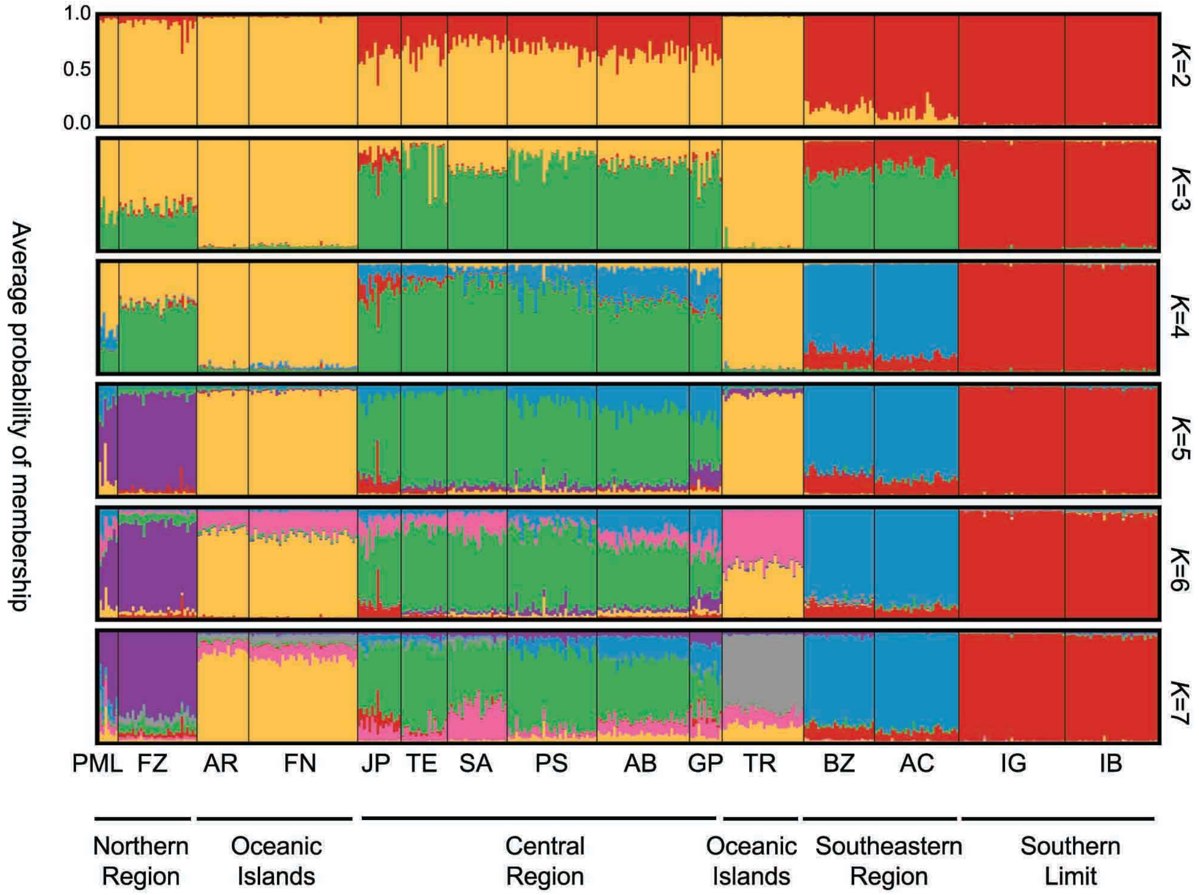

Figure S3: Bar plot showing the average probability of membership of each sample (vertical bars) in each population (colors) for *K*=2 of *Mussismilia hispida* individuals from Oceanic Islands' sites. Analyses were made with 10 iterations and without sampling locations as prior. Sampling sites are in the y-axis separated by vertical black lines, abbreviated as in Table 1.

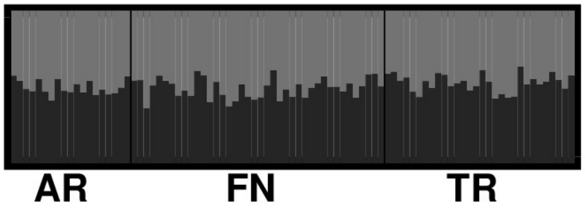

Supplement: Supplementary file 1 — Supplementary material [file 41598_2018_21010_MOESM1_ESM.pdf]
